# Analysis of the evidence-practice gap to facilitate proper medical care for the elderly: investigation, using databases, of utilization measures for National Database of Health Insurance Claims and Specific Health Checkups of Japan (NDB)

**DOI:** 10.1186/s12199-017-0644-5

**Published:** 2017-06-06

**Authors:** Takeo Nakayama, Yuichi Imanaka, Yasushi Okuno, Genta Kato, Tomohiro Kuroda, Rei Goto, Shiro Tanaka, Hiroshi Tamura, Shunichi Fukuhara, Shingo Fukuma, Manabu Muto, Motoko Yanagita, Yosuke Yamamoto, on behalf of BiDAME: Big Data Analysis of Medical Care for the Elderly in Kyoto, T. Funakoshi, S. Goto, E. Goto, N. Hanaki, S. Hiragi, T. Ikenoue, T. Iwao, K Kawakami, N. Kondo, S. Kunisawa, Y. Mori, M. Nakatsui, Y. Neff, S. Ohtera, K. Okamoto, T. Otsubo, H. Saito, Y. Saito, M. Sakai, I. Sato, K. Seto, S. Shimizu, Y. Takahashi, K. Yamashita, T. Yoshida

**Affiliations:** 1 Department of Health Informatics, Kyoto University, School of Public Health, Yoshida Konoe-cho, Sakyo-ku, Kyoto, 606-8501 Japan; 2 Department of Healthcare Economics and Quality Management, Kyoto University Graduate School of Medicine, School of Public Health, Kyoto, Japan; 3 Department of Biomedical Data Intelligence, Graduate School of Medicine, Kyoto University, Kyoto, Japan; 4 Solutions Center for Health Insurance Claims, Kyoto University Hospital, Kyoto, Japan; 5 Division of Medical Information Technology and Administration Planning, Kyoto University Hospital, Kyoto, Japan; 6 Graduate School of Business Administration, Keio University, Yokohama, Japan; 7 Faculty of Economics, Kyoto University, Kyoto, Japan; 8 Department of Clinical Biostatistics, Graduate School of Medicine, Kyoto University, Kyoto, Japan; 9 Department of Healthcare Epidemiology, School of Public Health in the Graduate School of Medicine, Kyoto University, Kyoto, Japan; 10 Department of Therapeutic Oncology, Graduate School of Medicine, Kyoto University, Kyoto, Japan; 11 Department of Nephrology, Graduate School of Medicine, Kyoto University, Kyoto, Japan

**Keywords:** Claims data, Database, Elderly, Evidence-practice gap, Validation

## Abstract

As Japan becomes a super-aging society, presentation of the best ways to provide medical care for the elderly, and the direction of that care, are important national issues. Elderly people have multi-morbidity with numerous medical conditions and use many medical resources for complex treatment patterns. This increases the likelihood of inappropriate medical practices and an evidence-practice gap. The present study aimed to: derive findings that are applicable to policy from an elucidation of the actual state of medical care for the elderly; establish a foundation for the utilization of National Database of Health Insurance Claims and Specific Health Checkups of Japan (NDB), and present measures for the utilization of existing databases in parallel with NDB validation.

Cross-sectional and retrospective cohort studies were conducted using the NDB built by the Ministry of Health, Labor and Welfare of Japan, private health insurance claims databases, and the Kyoto University Hospital database (including related hospitals). Medical practices (drug prescription, interventional procedures, testing) related to four issues—potential inappropriate medication, cancer therapy, chronic kidney disease treatment, and end-of-life care—will be described. The relationships between these issues and clinical outcomes (death, initiation of dialysis and other adverse events) will be evaluated, if possible.

## Background

As Japan becomes a super-aged society, the question of how medical care for the elderly is provided and the direction of that care are important national issues. Elderly people are physically frail and consequently often have multi-morbidity [1, 2], and thus, treatment patterns are often complex [3–5]. As a result, cases of polypharmacy in elderly people are not uncommon in clinical practice [6]. Treatment guidelines have been created with the aim of avoiding this kind of potentially inappropriate medical care, but those recommendations are not always properly implemented in clinical practice. The problem of when evidence from clinical research and treatment guidelines is not utilized in practice is called an evidence-practice gap [7]. In the USA, there are reports on the state of evidence-based health care [8] and proposals for narrowing the gap in actual clinical practice [9–11].

In Japan, the computerization of health and medical information is progressing, and large databases have been established. The utilization of these databases is an important issue. Since the mid-2000s, private companies have created databases of health insurance society claims that are used for research purposes [12, 13]. At the national level, National Database of Health Insurance Claims and Specific Health Checkups of Japan (NDB) has been constructed together with enforcement of the “Act on Assurance of Medical Care for Elderly People” of 2008. Since 2011, the NDB has been used secondarily for research purposes.

These databases are the quantitative information to measure the actual state of medical environment. There are few research papers which reported the actual situation of medical care in elderly based on a large-scale of data in Japan [14, 15], however, none of the papers which discuss about evidence-practice gap in elderly in super-aging society based on large-scale of data as NDB can be found currently.

In this study, we validate NDB in the onsite research center at Kyoto University Hospital, settled by the Ministry of Health, Labor and Welfare of Japan, to elucidate the actual state of medical care for the elderly and actual situation of evidence-practice gap. Research procedures are shown as follows.

First, we validate and estimate reliability of NDB using a database of the Kyoto University Hospital (including associated hospitals database), and private health insurance claims databases. Additionally, we compare the characteristic points of each database through validation and show the guidance for choosing the database depend on specific purpose.

Secondly, we elucidate how medical care for the elderly is provided in a super-aging society in Japan. To elucidate, the following three examples are settling: 1. potential inappropriate medication (PIM), 2. cancer treatment, 3. chronic kidney disease (CKD) treatment. PIM was chosen because improper use of medicine and medicine interaction caused by multiple drug administration cause adverse events and impair quality of life (QOL) of the elderly. We will conduct research that will contribute to safe medication for the elderly. The reason for choosing cancer treatment is that cancer is the disease of the main topic even in the world [16] and it is the leading cause of death in Japan, too [17]. The reason for choosing CKD treatment is one of the major complications of lifestyle-related diseases, and if it can delay progress by early intervention, and it will lead to improvement of QOL.

We will select databases which suit for elucidating the actual situation of these three examples based on selection guidelines by validation of NDB.

Third, through finding aspects, we will discuss the evidence-practice gap. Also, we will assess the examples in the view of medical economics, because Japan will enter the super-aged society ahead of the world, the elderly population will continue to increase [18] and medical costs will be expected to increase. We will clarify the current situation of medical costs and lead to the creation of solutions.

For “PIM”, we will clarify the status of drug prescriptions that should be limited in elderly people and polypharmacy, and show findings that will serve to alert medical practitioners to this. In “Cancer treatment”, we will check the implementation status of treatment recommendation guidelines, which will contribute to normalization of proper treatment and elimination of disparities, and to develop a system for the provision of novel cancer care. For “CKD treatment,” we will clarify the implementation status of existing treatment quality indices, provide information that will contribute to efforts to prevent increasing severity by the insurer, and assess the effects on health care costs.

Then, comprehensively considering the findings through efforts related to the three examples above, we will focus on “Medical care provided to end-of-life elderly patients”, because QOL of elderly patients is that concerns not only elderly patients but also medical state in super-aging society as a whole. We will show the actual palliative care and other end-of-life care that is currently provided, and build a foundation for social discussion of the kind of medical care that is desirable for elderly people.

Finally, from the above, we will elucidate the current state of evidence-practice gap from the perspectives of both under- and over-treatment in the super-aging society of Japan, and analyze medical economically, together with presentation of ways to provide medical care in the future and directions for this care in aspect of epidemiology.

## Methods

### Materials

In Japan, all citizens are generally covered with public health insurance [19] as a whole.

**Fig. 1:**
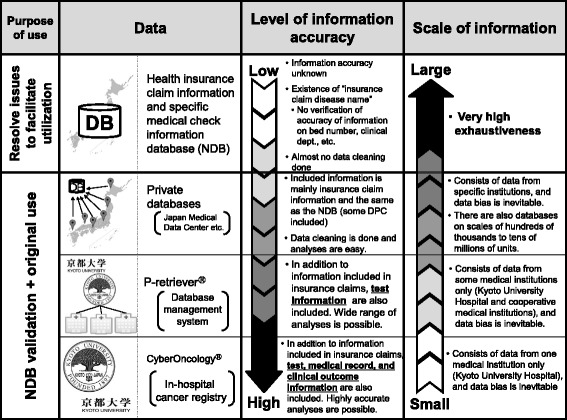
Characteristics of the data used in this study

In this study, the research questions will be analyzed using the data in the NDB, which holds billing information on more than 95% of the all current health insurance treatments under public health insurance system, Kyoto University Hospital databases including associated hospitals databases (CyberOncology®, p-Retriever®), and private databases shown as Fig. 1. NDB is a health insurance claims information and specific medical checkups information database. CyberOncology® is an in-hospital registry including insurance claims, test information, medical record, and clinical outcome information, that highly accurate analyses are possible. p-Retriever® is a database management system, including insurance claims and test information, which is possible to analyze highly in wide range. Data that are suitable for achieving the aims of this study will be extracted from NDB. Both the NDB and private data consist mainly of health insurance claims, including basic patient information such as sex and age plus things such as the number of insurance points, the name of the illness or injury, medical practice information, and drug administration and prescription information. Information that is not included in health insurance claims data will be assessed in detail with complementary use of Kyoto University Hospital databases.

### Study structure

This study is planned and implemented by a steering committee consisting of individuals from the Kyoto University Graduate School of Medicine, Kyoto University Hospital, and Division of Medical Information Technology and Administration Planning in Kyoto University Hospital.

### Schedule

**Fig. 2:**
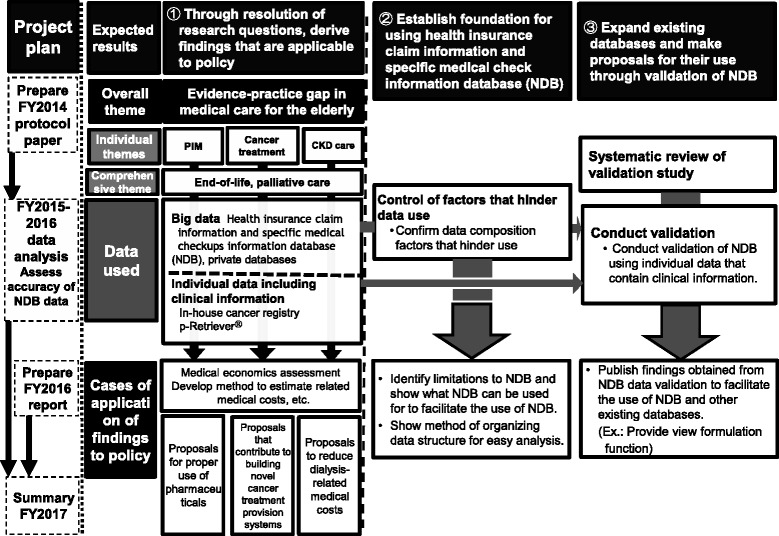
Study flow diagram

The study was prepared in 2014 and began in April 2015 and will continue until March 2017 (Fig. 2). The first fiscal year (2015) is centered on accumulating previous findings on the research questions, developing database systems, systemic review of validation studies, tentative analysis of the databases, and establishing an operational structure, environment, and regulations for the NDB onsite research center. In the second fiscal year (2016), analysis of the particulars of the NDB and other databases will be conducted and the direction for policy application will be investigated.

## Instruments

### Methods for validation

In secondary use of medical data, validation studies are recommended to assess the accuracy of definitions of things such as exposure and outcomes used in the study. Validation refers to comparison of the validity of information included in the data in question with other highly reliable information sources in secondary use when performing an investigation. Health insurance claims do not include test results, and the name of the disease does not necessarily reflect the patient’s medical diagnosis due to insurance claims needs. Thus, caution is needed in analysis for research purposes since the “insurance claims disease name” is included. Health care data included in hospital databases includes test values, and since a more detailed picture of the patient can be obtained, the two need to be compared in validation as a reference to assess the validity of the health insurance claims disease name. Prior to the actual validation study in this study, a systematic review will be conducted of existing research on the validation of health insurance claims data.

### Three examples to elucidate the actual state of medical care for the elderly

To elucidate the actual state of medical care for the elderly, this study has the three examples which are (1) potential inappropriate medication (PIM), (2) cancer treatment, (3) chronic kidney disease (CKD)treatment. The current state of each example will be clarified below mentioned specified criteria based on NDB. After all, through all findings, the evidence-practice gap for elderly will be discussed and assessed medical economically.

#### PIM

##### Elucidation of actual state of medical care for PIM

The evidence to clarify the state of evidence-practice gap is STOPP (Screening Tool of Older person’s Potentially inappropriate Prescriptions) list of the Japan Geriatrics Society guidelines and the Japanese version of the Beers criteria [20–23]. We will specify drugs that are preferably avoided in elderly people following STOPP list and Beers criteria through NDB, based on the frequency of use of these drugs (number of prescriptions, dosage, concomitant medications including polypharmacy, etc.), incidence of new administration, related factors and association with adverse events (verification centered on diseases included in the Charlson Comorbidity Index). These findings will be analyzed by strata including sex, age, comorbidities, and place of treatment.

##### Medical economics assessments

Database of the patients aged 70 will be focused on as that is when the individual payment rate increases from 30% to 10 or 20%, depends on the income, and the effect of the change in the individual payment rate on medical consultations and prescription behavior is analyzed.

#### Cancer treatment

##### Elucidation of actual state of medical care for cancer treatment

Since cancer is the leading cause of death in Japan [17], we focus on the actual medical state of cancer treatment. In the 5 major cancer disease in Japan (breast, colon and rectum, stomach, lung, liver), we will choose 3 kinds of cancer (stomach [24], colon and rectum [25], liver [26]). Checking the implementation status of treatment recommendation guidelines of 3 kinds of cancer [24–26] as criteria, following points will be elucidated; treatment patterns (operative procedure, anti-cancer agent prescription), implementation of standard treatment, occurrence of adverse events, treatment patterns that lead to better treatment efficacy and fewer adverse events, and medical costs analyzed by strata for age, presence of comorbidity, region, hospital function, and hospital size. The Kyoto University Hospital database, CyberOncology®, which consists of treatment information which is not included in health insurance claims will be used for validation.

##### Medical economics assessments

In a medical economics assessment, a regression analysis is conducted to see how much of the variation in medical costs can be explained by age, sex, and treatment pattern.

#### CKD treatment

##### Elucidation of actual state of medical care for CKD treatment

In Japan, Statistically, the total number of patients with CKD is about 240,000 and the number of dialysis patients is 320,000, approximately 70% of dialysis patients occupied by over 65 years in 2015 [27]. Based on 11 different quality indices named Quality Indicators for Chronic Kidney Disease self-developed between 2010 and 2013 [28], we will choose seven different quality indices [ use of Renin-Angiotensin System (RAS) inhibitors, serum creatinine measurements in patients using RAS inhibitors, serum potassium measurements in patients using RAS inhibitors, fluid replacement in patients undergoing contrast-enhanced computerized tomography (CT), avoidance of regular use of Non-Steroidal Anti-Inflammatory Drugs (NSAIDs), implementation of nutritional counseling, implementation of urinalysis [28–30] ] obtained from health insurance claims information will be measured. Treatment quality and associations with the start of dialysis treatment will be investigated longitudinally. Health insurance claims information at hospitals associated with Kyoto University that have introduced p-Retriever®, which can extract health insurance claims and test values, will be used for validation.

##### Medical economics assessment

In a medical economics assessment, the effects of differences in treatment quality on patient symptoms are modeled and the effect of treatment quality on cost-effectiveness is evaluated.

#### Medical care given to end-of-life elderly patients

##### Elucidation of actual state of medical care given to end-of-life elderly patients

Through NDB and private database, we will focus on the data of elderly of more than 65 years old [31, 32], and going backward from death from one month to one year, and clarified the content, frequency, and cost of medical care provided in the end-of-life stage. Prescriptions and implementation status are examined from the perspectives of both under- and over-treatment, particularly cardiopulmonary resuscitation, gastric fistula, blood transfusion, dementia medications, antihypertensive medication, cholesterol-lowering drugs, hypoglycemic agents, osteoporosis drugs, anticancer agents, and palliative care. In health insurance claims, there is an “outcome” column, but it is not always filled in. When death as an outcome cannot be ascertained, the operational definition of a judgment of “death” in health insurance data from the suspension of treatment that had been continued up to that point is investigated. In parallel with an understanding of the end-of-life period retroactively from death, the actual medical behaviors above are determined with elderly people divided into age cohorts.

##### Medical Economics Assessment

In a medical economics assessment, the medical care and the associated costs are investigated by region, by the type of care (hospital or home care), and by the type of disease (cancer, stroke, ischemic heart disease and dementia etc.).

## Strengths/Limitations

The NDB contains exhaustive data including information on nearly all health insurance claims. However, the state of insurance claims information, such as disease name and other factors, do not reflect actual clinical status of patients. A number of previous studies have indicated that validation is a common issue worldwide when using databases, but there are almost no reports from Japan. The significance of this study is in raising the validity of findings obtained by carrying out validation studies, and in the suggestions it provides for precautions and further potential uses of health insurance claims information and specific medical checkups information and the NDB, in which such data is accumulated. At the same time, the study is limited by the fact that the NDB itself cannot be linked to other data, accordingly, the NDB cannot be directly validated, and comparisons with other health care information, such as insurance claims information and test values, must be done within limited groups, such as several medical institutions.

## Abbreviations

BiDAME: Big Data Analysis of Medical care for the Elderly in Kyoto
CKD: Chronic kidney disease
CT: Computerized tomography
NDB: National Database of Health Insurance Claims and Specific Health Checkups of Japan
NSAIDs: Non-Steroidal Anti-Inflammatory Drugs
PIM: Potential inappropriate medication
QOL: Quality of life
RAS: Renin-Angiotenshin System
STOPP: Screening Tool of Older person’s Potentially inappropriate Prescriptions
